# 46,XY female sex reversal syndrome with bilateral gonadoblastoma and dysgerminoma

**DOI:** 10.3892/etm.2014.1922

**Published:** 2014-08-19

**Authors:** XUE DU, XUHONG ZHANG, YONGMEI LI, YUKUN HAN

**Affiliations:** 1 Department of Obstetrics and Gynecology, General Hospital, Tianjin Medical University, Tianjin 300052, P.R. China; 2 Department of Microbiology and Immunology, Tianjin Medical University, Tianjin 300070, P.R. China

**Keywords:** sex reversal syndrome, simple gonadal dysgenesis, gonadoblastoma, dysgerminoma, sex determining region Y gene

## Abstract

Sex reversal syndrome is a rare congenital condition of complete or disordered gonadal development leading to discordance between the genetic, gonadal and phenotypic sexes, including 46,XX and 46,XY. The gonadoblastoma on the Y-chromosome (GBY) region is associated with an increased risk of developing type II germ cell tumors/cancer. The present study reports a unique case of a phenotypically normal female (age 17 years), presenting with primary amenorrhea and later diagnosed with 46,XY female sex reversal syndrome. Following bilateral gonadectomy, bilateral gonadoblastoma and dysgerminoma were diagnosed. Thus, estrogen replacement therapy was administered periodically to promote the development of secondary sexual characteristics and menstruation, and to prevent osteoporosis. A four year follow-up showed no tumor recurrence and a regular menstrual cycle in this patient.

## Introduction

The 46,XY sex reversal syndrome (SRS) is characterized by a 46,XY karyotype, normal female external genitalia, completely undeveloped (‘streak’) gonads, no sperm production and the presence of normal Müllerian structures. The syndrome occurs with an estimated incidence of 1:5,000 (1). Individuals with an underlying SRS, particularly those with specific Y chromosomal material in their karyotype, have an increased risk of developing a type II germ cell tumor/cancer (GCC) (1). GCCs arise from primordial germ cells or gonocytes and may be subdivided into seminomas/dysgerminomas and non-seminomas with carcinoma *in situ* or gonadoblastomas as precursor lesions (2). This study presents a unique case with bilateral gonadoblastoma and dysgerminoma in a girl presenting with primary amenorrhea at the age of 17 years, who was initially diagnosed with 46,XY SRS.

## Case report

A 17-year-old girl was admitted to the General Hospital of Tianjin Medical University (Tianjin, China) with no menarche and continuous height growth. The patient was treated with artificial estrogen and progesterone to induce an artificial menstrual cycle for two months, but only a small amount of pink secretion was observed in the vagina. The patient was born following a full-term normal delivery to non-consanguineous parents, and her mother denied the use of any sex hormone drugs or exposure to radioactive substances during pregnancy. The younger brother of the patient exhibited a normal phenotype. Physical examination showed that the patient had relatively long upper extremities, and the arm span was greater than the height of the patient. The patient exhibited a female appearance and voice, with little subcutaneous fat, no beard or laryngeal prominence, and hypoplastic breasts with a light areola. No palpable mass was identified in the groin or labia majora. The patient exhibited female external genitalia, with normal labia majora and minora, sparse pubic hair and a visible vaginal orifice. The anal examination showed a small uterus, a mass of ~4.0×3.0 cm in the right adnexa, and another mass of ~3.0×3.0 cm in the left adnexa, both of which were hard and mobile. The B-mode ultrasound showed an infantile uterus, the endometrium appeared as a thin echogenic line, and the masses in the adnexa were non-homogeneous measuring ~45×15 mm (right) and ~33×14 mm (left), respectively. Serum sex hormone analysis revealed that the follicle-stimulating hormone level was 104.5 IU/l, the luteinizing hormone level was 43.1 IU/l, the estradiol level was <10 pg/ml and the testosterone level was 55 ng/dl. The patient exhibited a slightly higher level of serum testosterone than normal and a low level of serum estrogen. Peripheral blood chromosome analysis showed the 46,XY karyotype. No gene deletions were detected at sY84, sY86, sY127, sY134, sY254 and sY255 of the sex determining region Y (*SRY*) gene. The patient was diagnosed with 46,XY female SRS, simple gonadal dysgenesis and unclear bilateral gonadal mass.

During the laparotomy, the small uterus was apparent with a size of 5.0×5.0 cm. The right gonad measured 4.0×3.0×3.0 cm, had an ovary-like appearance with a complete capsule, and was white, smooth and hard in texture; while the left gonad measured 3.0×3.0×3.0 cm and had a similar appearance to the right gonad. The uterine tubes were narrow. No testes or associated tissues were identified in the region between the superficial and deep inguinal rings and the urinary bladder, and the bifurcation of the common iliac artery. Bilateral gonadectomy was performed for simple gonadal dysgenesis. The patient was then pathologically diagnosed with bilateral ovarian gonadoblastoma and right dysgerminoma. The patient was genetically male due to her 46,XY karyotype, but socially and psychologically female in every respect; thus, an important aspect of postoperative treatment was to maintain the female characteristics. Estrogen (Premarin) and medroxyprogesterone acetate replacement therapy was administered. A four year follow-up showed that the patient treated with a sequential therapy of estrogen and progesterone had a regular menstrual cycle and no tumor recurrence.

This study was approved by the Ethics committee of Tianjin Medical University (Tianjin, China) and patient informed consent was obtained.

## Discussion

Although the genetic sex of an individual is determined at fertilization, it is at the embryonic stage that the sexual differentiation of the reproductive system begins. The primordial gonad is bipotential and can differentiate into a testis or an ovary, depending on the *SRY* gene located in the short arm of the Y-chromosome. The absence of *SRY* permits the bipotential gonad to differentiate into an ovary at the eighth week of the embryo, leading to the female phenotype. The mutation, deletion or translocation of *SRY* can affect the binding of the SRY proteins with DNA, and consequently contribute to sex reversal (3). In the present case, the patient with a positive *SRY* gene on the Y-chromosome had no testes, but had an ovarian cortex and hypoplastic female external genitalia. This finding indicates that the *SRY* gene is not the only gene responsible for testis development, and there may be other genes besides the *SRY* gene involved as well (4). Duplication of the DAX1 (also known as *NR0B1*) and *WNT4* genes, as well as haploinsufficiency of the *SOX9*, *SF1*, *WT1* and *DMRT1*-*DMRT2* genes, have been considered responsible for the development of 46,XY sex reversal (5,6) and therefore the *SRY* gene does not play a significant role in the etiology of the disease in this case. No gene deletions including sY84, sY86, sY127, sY134, sY254 and sY255 loci of the *SRY* gene were detected; future studies are required to focus on other loci of the *SRY* gene. Although no genetic abnormality was detected in this patient, there may be abnormalities in the content and function of SRY protein, which may result in the lack of testis development and the lack of secretion of testosterone and Müllerian-inhibiting factor. As a result, the Wolffian duct degenerates and does not differentiate into the male reproductive tract. By contrast, in the absence of Müllerian-inhibiting factor, the Müllerian duct differentiates into the oviducts, uterus, cervix and upper vagina. However, such female patients without the presence of XX chromosomes do not have a properly developed uterus or ovary, which is characterized by primary amenorrhea, high height, poorly developed secondary sex characteristics and external genitalia. The discordance between chromosomal and gonadal sex is determined as SRS (4), including 46,XX male and 46,XY female SRS.

The undeveloped primordial gonad does not have a normal physiological function, and thus has a potential risk of malignancy. Patients with chromosomal disorders of sex development (DSD) that have specific Y-chromosomal material in their karyotype, the gonadoblastoma on the Y-chromosome (GBY) region, have an increased risk of developing GCC (7). GCC risk varies, but is estimated to be >30% in patients with simple gonadal dysgenesis and is often bilateral (8).

**Figure 1:**
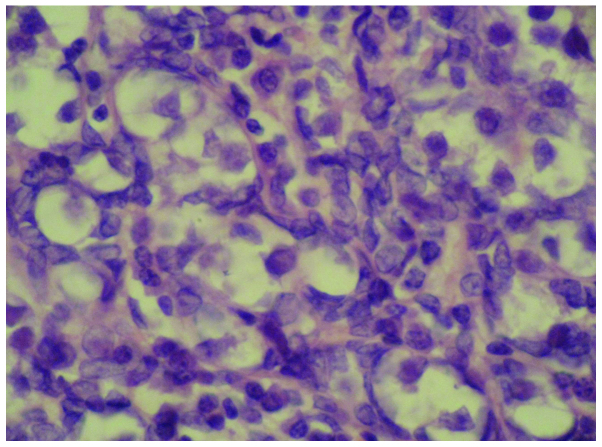
Gonadoblastoma (magnification, ×400; hematoxylin and eosin staining). The tumor cells, round or ovoid in shape, form nests that vary greatly in size. The nests are surrounded by fibrous connective tissue and have distinct borders. At the center of the nest are large and round germ cells with abundant transparent cytoplasm and deeply stained nucleus. The supporting cells and granular cells, small in size and spindle or ovoid in shape, are arranged in clusters around the nests.

**Figure 2:**
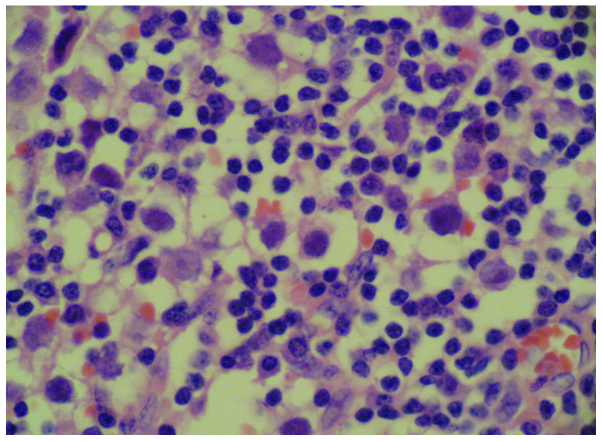
Dysgerminoma (magnification, ×400, hematoxylin and eosin staining). The tumor cells are large in size and round or ovoid in shape, and have distinct borders. The nucleus at the center of the cell is large and round, and nuclear division is often observed. There is abundant transparent cytoplasm. Lymphocyte infiltration is observed in the connective tissue.

GCC may be induced by the interaction of abnormal streak gonads with the intra-abdominal environment, or by gene mutation. As a result, gonadectomy should be performed on the diseased side or bilaterally for prophylactic purposes. In the present case, the patient received bilateral gonadectomy. Fig. 1 shows the pathological features of the ovarian gonadoblastoma: the supporting cells and granular cells are arranged around the gonadoblastoma nests, and the mixed cells are at the center of the nests. It is also noted that the intercellular substance is calcified, and thus the section is off-white or grayish yellow, like sandstone. Gonadoblastoma can be either in a pure form or mixed with other germ cell tumors. In 50–60% of cases gonadoblastomas are associated with malignant germ cell tumours, most presented as pure dysgerminom or less frequently as immature teratoma, Yolk sac tumour (9). The mixed gonadoblastoma is potentially malignant and has a poor prognosis. In the present case, the patient was diagnosed as having stage-Ia gonadoblastoma mixed with malignant dysgerminoma in the right ovary, and pure benign gonadoblastoma in the left ovary, as shown in Fig. 2. Follow-up was performed at a fixed interval. In this case, although the chromosomal sex of the SRS patient was male, the patient had female external genitalia and had been living as a female in the preceding years. Thus, estrogen replacement therapy was administered periodically to promote the development of secondary sexual characteristics and menstruation, and at the same time, measures were taken to prevent osteoporosis. It allowed the patient to have a more regular menstrual cycle, and the four year follow-up showed no tumor recurrence.
